# Temperature and Precipitation More Than Tree Cover Affect the Distribution Patterns of Epiphytic Mosses within the *Orthotrichaceae* Family in China and Adjacent Areas

**DOI:** 10.3390/plants12010222

**Published:** 2023-01-03

**Authors:** Lucie Fialová, Vítězslav Plášek, Ewelina Klichowska, Shuiliang Guo, Marcin Nobis

**Affiliations:** 1 Department of Botany, University of Ostrava, Chittussiho 10, 710 00 Ostrava, Czech Republic; 2 Institute of Biology, University of Opole, Oleska 48, 45-052 Opole, Poland; 3 Institute of Botany, Faculty of Biology, Jagiellonian University, Gronstajowa 3, 30-387 Kraków, Poland; 4 College of Life and Environmental Sciences, Shanghai Normal University, 100 Guilin Road, Shanghai 200234, China

**Keywords:** Asia, bryophytes, distribution maps, ecological niche modelling, *Leratia*, *Lewinskya*, *Macromitrium*, *Nyholmiella*, *Orthotrichum*, *Ulota*

## Abstract

Epiphytes, including vascular and non-vascular, constitute a large part of global plant biodiversity. Distribution of obligatory epiphytic bryophytes results from climate and local habitat conditions. The most important epiphytic bryophytes and at the same time poorly investigated and taxonomically problematic ones belong to the family Orthotrichaceae. Epiphytic mosses are also ideal organisms for species modelling, because of having no roots, they are highly dependent on external environmental conditions. For this purpose, we used the ecological niche modelling approach to define their potential distribution in China and adjacent areas and explore factors that shape this distribution. We used 617 occurrence records of 23 species from six genera within the Orthotrichaceae family. Our results suggest that the distribution of members of the Orthotrichaceae family is predominantly affected by bioclimatic variables, especially bio10 (mean temperature of the warmest quarter), bio15 (precipitation seasonality), bio18 (precipitation of the warmest quarter), bio19 (precipitation of the coldest quarter), bio9 (mean temperature of the driest quarter), and bio2 (mean diurnal range). However, the distribution of particular genera is ruled by a different set of those variables. The distribution of two genera (*Leratia* and *Ulota*) is also highly influenced by land cover (especially mixed/other trees), whereas human footprint shows a moderate contribution to models of three genera (*Lewinskya*, *Orthotrichum*, *Nyholmiella*). Based on the occupied climatic niche and distribution patterns, representatives of the studied family are divided into two groups. The ‘*western-montane* group‘ is characterised by lower temperatures and lower precipitation whereas the ‘*eastern-lowland*’ group‘ by more humid and warmer conditions.

## 1. Introduction

The distribution of natural vegetation at large spatial scales is directly impacted by climate [1,2,3]. However, potential drivers of species-richness patterns that have also been considered are environmental factors such as water availability and habitat heterogeneity [4,5,6,7,8]. In the case of obligatory epiphytic bryophytes, patterns of distribution are the results of the relationship between climate and specific environments [9,10,11,12]. Epiphytic bryophytes are generally willing to occupy the bark of trees both in temperate forests and in more humid tropical areas [13]. With approximately 850 widely distributed subcosmopolitan species, the family Orthotrichaceae [14,15,16,17,18,19,20,21,22] is one of the main components of the cryptogam epiphytic communities and occurs on tree trunks and branches, and occasionally they are found also in saxicolous habitats. In Central and East Asia, the family is represented by eight genera, namely *Leratia*, *Lewinskya*, *Macrocoma*, *Macromitrium*, *Nyholmiella*, *Orthotrichum*, *Ulota,* and *Zygodon*, which can be found in most biomes, from tundra to wet tropical forests. Representatives of *Macromitrium* and partly also *Ulota* are associated with more humid tropical areas and are characterised by resistance to strong competition [23,24]. Whereas the other epiphytic taxa of the family, which are less steady competitors in comparison to other species of bryophytes and lichens, prefer rather sparse forests, bushes, and also alleys or solitairy trees that are exposed to harsh, less humid, or even semi-dry climatic conditions [11,12,25,26,27,28]. Therefore, species within such genera as *Macromitrium* and *Leratia* are very often observed in the south-eastern part of China [23,24,29,30], where the climate is warmer and more humid, and the temperature difference between day and night smaller [31], whereas representatives of *Lewinskya*, *Nyholmiella*, and *Orthotrichum* are much more frequently observed in the mountain areas in the central and western part of the country [32,33,34]. This observation is related to the distribution of climatic zones (wet and warm vs. dry and cold) in China and generally in eastern Asia [31,35,36].

Ecological niche modelling (ENM) is a widespread method to determine species distribution patterns. In simple terms, ENM uses computer algorithms to predict the distributions of species across geographic space and time by using occurrence data and environmental data. ENM estimates the relationship between species records at sites and the environmental and/or spatial characteristics of those sites [37]. Despite the frequent use of this method, and despite the fact that mosses are an ideal model organism for species modelling because, having no roots, they are highly dependent on external environmental conditions, the general patterns of distribution of epiphytic mosses from the Orthotrichaceae family are still underexplored.

In this paper, we aim to: (i) investigate macroscale factors affecting the distribution of epiphytic mosses from the family Orthotrichaceae in Central and East Asia; (ii) find the differences in ecological niches occupied by representatives of the family Orthotrichaceae; and (iii) assess the impact of anthropopressure on species distribution patterns. The results can help to define factors influencing the geographical distribution patterns of the Orthotrichaceae family and point out hotspots of the highest epiphyte diversity and at the same time the most important areas for conservation purposes.

## 2. Materials and Methods

### 2.1. Characteristics of the Studied Genera

In this study, distribution data of species from six genera within the Orthotrichaceae family (*Leratia*, *Lewinskya*, *Macromitrium*, *Nyholmiella*, *Orthotrichum,* and *Ulota*) were evaluated. Brief descriptions of the genera and their taxonomic distinctness are explained below. The genera *Macrocoma* and *Zygodon*, which also occur in the study area, were not included in the modelling, as their taxonomic revision is still ongoing and detailed data on the distribution of particular species was not available.

#### 2.1.1. *Leratia* Broth. & Paris

The taxonomy of this genus was not completely clear in the past. It includes three very geographically isolated species. Goffinet et al. [15], using molecular data, clearly proved that the Asian and Australasian *Leratia obtusifolia*, North American *L. exigua* and New Caledonian *L. neocaledonica* belong to an isolated and strongly supported clade, to which they applied the generic name *Leratia*. Only *Leratia exigua* was used for modelling, as it is the only representative of this genus occurring in China and other parts of the Asian continent [34].

*Leratia exigua* can be distinguished from similar orthotrichaceous mosses by a combination of features: decurrent leaves with plane margins; superficial stomata and hyaline; and keeled endostome segments [38].

#### 2.1.2. *Lewinskya* F.Lara, Garilleti & Goffinet

The genus *Lewinskya* was described as a result of taxonomic and molecular studies that confirmed polyphyly of the broadly conceived traditional genus *Orthotrichum* s.l. Three segregates have been split from it, including *Lewinskya*, *Nyholmiella*, and *Pulvigera* [19,39,40,41]. Species of the genus *Lewinskya* differ from the representatives of the genus *Orthotrichum* mainly in the presence of superficial stomata [19]. Currently, the genus *Lewinskya* comprises 70 taxa worldwide [41], of which 14 *Lewinskya* species and two varieties have been recorded in China [34]. Here for the purpose of distribution modelling, we used data for the six most common species, namely *L. affinis*, *L. exigua*, *L. hookeri*, *L. speciosa*, *L. striata*, and *L. vladikavkana*.

#### 2.1.3. *Macromitrium* Brid

Among the epiphytic bryophytes, *Macromitrium* is considered one of the largest moss genera in the world with the majority of its species distributed in tropical and subtropical regions [42,43]. According to Vitt and Ramsay [44], there are 350 species worldwide but only 128 can be morphologically differentiated [42]. In China, there are 17 species and one variety for which occurrence was recently confirmed [38]; however, for the purpose of distribution modelling we used data of the two most common species, *M. cavaleriei* and *M. gymnostomum*.

#### 2.1.4. *Nyholmiella* Holmen & Warncke

The taxonomic position of the genus *Nyholmiella* has been uncertain in the past and it has been alternately classified as either part of the widely conceived genus *Orthotrichum* or as a separate genus [45,46]. Sawicki et al. [17] proposed the first systematic consequences since 2004 to reflect phylogenetic reconstruction, and reinstated the name *Nyholmiella*. Currently, the genus *Nyholmiella* comprises 3 taxa worldwide [17,47]. Only *N. obtusifolia* was used for distribution modelling, as it is the only representative of this genus occurring in China and adjacent areas [34]. Historically, the species *N. gymnostoma* was also reported from the territory of China [48], but after a taxonomic revision it was proved to be *N. obtusifolia*, and consequently, *N. gymnostoma* has been excluded from the list of Chinese bryophytes [34].

#### 2.1.5. *Orthotrichum* Hedw

The genus *Orthotrichum* is the only orthotrichaceaous moss—of those which occurred in the study area—with immersed stomata [39]. According to Lara et al. [41], the genus comprises 103 taxa worldwide, of which the Northern Hemisphere hosts 83%. In the course of a new taxonomical revision, 29 species and two varieties have been reported from China [34]. Considering that the genus is the most numerous group of all orthotrichaceous mosses in China and the surrounding Asian countries, we used 14 representatives for distribution modelling, namely *Orthotrichum alpestre*, *O. anomalum*, *O. callistomum*, *O. consobrinum*, *O. crenulatum*, *O. crispifolium*, *O. dasymitrium*, *O. griffithii*, *O. pallens*, *O. pamiricum*, *O. pumilum*, *O. scanicum*, *O. sordidum*, and *O. urnigerum*.

#### 2.1.6. *Ulota* D.Mohr

Recent taxonomic studies have shown that the genus *Ulota* is polyphyletic [19,21,22]. First, based on molecular studies, *Ulota phyllantha* was displaced into the new genus *Plenogemma* [19] and latterly *U. bellii* into the genus *Rehubryum* [22]. According to Wang and Jia [49], the genus *Ulota* s. str. comprises about 50 taxa worldwide.

Species of *Ulota* are distinguished from other orthotrichaceous mosses based on a combination of characters—crisped to slightly twisted leaves (when dry) and leaf base with a well-differentiated border formed by one or a few rows of quadrate to rectangular hyaline cells [39]. The occurrence of eight species is recently known from China [29,38]. Distribution data of six of them, namely *U. crispa*, *U. gigantospora*, *U. gymnostoma*, *U. perbreviseta*, *U. robusta*, and *U. yunnanensis* were used here for modelling.

### 2.2. Occurrence Data

The data on the distribution of species that were used for modelling comes from our own collections, a query of the literature data, and also from the revision of the moss specimens housed in major European and Chinese herbaria. Field research in twelve provinces of China, including Anhui, Guizhou, Henan, Hubei, Jiangsu, Shaanxi, Shanxi, Shanghai, Sichuan, Xinjiang, Yunnan, and Zhejiang were carried out in 2015–2019. In addition, the authors carried out several bryological expeditions to Central Asia (Tajikistan, Kirgizstan, Kazakhstan, and Afghanistan), where the Orthotrichaceae family was studied in detail in the years 2011–2015. A complete revision of herbarium items of epiphytic bryophytes was also carried out in the herbarium of the Botanical Institute of the Tajikistan Academy of Sciences in Dushanbe.

During the study, the herbarium collections from the following Chinese and European herbaria have been revised (acronyms of the herbaria according to *Index herbariorum* but for some Chinese herbaria they are unauthorised and introduced only for the purposes of the present article; they are distinguished by not using bold letters):

**C**—University of Copenhagen, Denmark;

**E**—Royal Botanic Garden Edinburgh, Scotland, U.K;

GuZU—Guizhou University, China;

**H**—University of Helsinky, Finland;

**HSNU**—Herbaria of East China Normal University, China;

HuBU—Hubei University, China;

**IFP**—Institute of Applied Ecology, Academia Sinica, Shenyang, China;

InMU—Inner Mongolia University, China;

**KRA—**Herbarium of the Inst. of Botany, Jagiellonian University, Kraków, Poland;

**KRAM**—Polish Academy of Sciences, Krakow, Poland;

**KUN**—Herbarium of Kunming Inst. of Botany, the Chinese Academy of Sci., China;

**OSTR**—University of Ostrava, Czech Republic;

**PC**—Muséum National d’Historie Naturelle, Paris, France;

**PE**—Institute of Botany, Chinese Academy of Science, Beijing, China;

ShM—Shanghai Museum, China;

ShU—Shanghai Normal University, China;

**TAD—**Botanical Inst. of the Tajikistan Academy of Sciences in Dushanbe, Tajikistan;

**W**—Naturhistorisches Museum, Wien, Austria;

**XJU**—Xinjiang University, China.

Because the Orthotrichaceae family is considered to be a taxonomically problematic group, whose determination is not elementary, we decided to use mainly distribution data from our own field research (33.8%), as well as revised herbarium specimens (64.4%), supplemented only in a small percentage by records from online databases (1.62%) and distribution data from published papers (0.18%). To prevent possible misidentification of taxa, all available individuals were morphologically determined or re-determined in the BRYOLAB laboratory (part of the Department of Biology and Ecology, at the University of Ostrava).

In summary, 2717 geographic occurrence records were collected from the area of Central Asia (e.g., Afghanistan, Kazakhstan, Kyrgyzstan, and Tajikistan), East and Southeast Asia (e.g., China, Japan, Mongolia, Taiwan, and Vietnam). To avoid problems resulting from inaccurate descriptions of localities we excluded from analysis data with missing geographic coordinates as well as data collected before 1960 [50]. To remove sampling bias, we first deleted duplicate records and then filtered the data through a grid with a WGS84 format and resolution of cells 0.1 × 0.1 -degree, as described in Hijmans et al. [51]. For filtering data, we used a sample function from dismo package in R software [52]. Preparation of occurrence data was performed using the open source software QGIS [53]. As a result, we obtained 617 occurrence records for the whole family Orthotrichaceae, including *Leratia* (22), *Lewinskya* (312), *Macromitrium* (110), *Nyholmiella* (53), *Orthotrichum* (354), and *Ulota* (19).

In addition to data analysis for individual genera, we also evaluated two ecological groups of mosses connected with different distribution patterns: and *western-montane* epiphytic group (479 records) and *eastern-lowland* epiphytic group (191 records). Based on distribution records we included *Lewinskya affinis*, *L. hookeri*, *L. speciosa*, *L. vladikavkana*, *Nyholmiella obtusifolia*, *Orthotrichum alpestre*, *O. anomalum*, *O. callistomum*, *O. crenulatum*, *O. dasymitrium*, *O. pallens*, *O. pamiricum*, *O. scanicum*, *O. sordidum*, *O. urnigerum*, and *Ulota robusta* in the *western-montane* epiphytic group, whereas *Leratia exigua*, *Macromitrium gymnostomum*, *M. cavaleriei*, *O. consobrinum*, *O. crispifolium*, *O. griffithii*, and *Ulota gymnostoma* were included in the *eastern-lowland* epiphytic group. In the case of species with wide distributions, the species were assigned to a particular group depending on their frequency (the majority of localities).

Nomenclature follows Lara et al. [41] and Sawicki et al. [19]. Duplicates of all herbarium specimens together with our own materials collected during field research in Central Asia and China are deposited in OSTR and KRAM.

### 2.3. Ecological Niche Modelling

For modelling the potential distribution of epiphytic mosses from the family Ortotrichaceae we used MaxEnt software 3.3.3, which is a generative species distribution modelling approach recommended for applications involving presence-only datasets and environmental predictors [54]. For our purpose, 25 variables of continuous environmental predictors, including 19 bioclimatic variables from WorldClim v.2.1 [55], 5 global 1-km consensus land cover v.2.2 [56], and human footprint v.3 [57] were initially used (Table 1). All variables were evaluated with distribution data for the entire *Orthotrichaceae* family. All environmental data were derived with a spatial resolution of 30 arcsec (approx. 1 km). Because one of the aims of the research was to create a general model of the distribution of epiphytic mosses of the Orthotrichaceae family in the region of Asia, the background area was defined by the geographic boundaries of Afghanistan, Bhutan, Cambodia, China, India, Iran, Japan, Kazakhstan, Kyrgyzstan, Laos, Mongolia, Myanmar, Nepal, North Korea, Pakistan, Philippines, South Korea, Taiwan, Tajikistan, Thailand, Turkmenistan, Uzbekistan, and Vietnam [58]. The number of background points was set to 10,000 (default value) [59].

Environmental covariates were clipped according to the mask and resampled to the same resolution and projection as the bioclimatic variables using the extract by mask function in ArcMap 10.5 software [60]. For each layer, the WGS-84 geographical coordinate system was chosen.

To avoid overfitting of the model we built a correlation matrix (Pearson’s correlation coefficient) and removed highly correlated variables (r > 0.7) [61], by generating a Pearson correlation matrix in Statistica software [62]. To choose which of the strongly correlated variables to remove, we performed a jackknife test of variable importance [63] and compared the percent contribution of the variables. We chose variables that showed higher positive gain values and higher percent contribution or those whose impact was easier to explain from a species biology point of view. For the final models, we used 15 variables, including 9 bioclimatic variables, human footprint, and 5 landcover classes (Table 1).

We ran models with default values (a maximum of 500 iterations, convergence threshold 0.00001, and five auto feature classes). We decided to choose a logistic format, as it is currently considered easier and potentially more accurate for interpretation than the cumulative and raw approaches [59,63]. The model was calibrated using 75% of the occurrence records and tested on the remaining 25%. We performed 20 replicates using the subsample replicated run type and then averaged the results. To provide a different random test/train partition in each replicate, we used the ‘random seed’ option. Because the distribution data came from different sources with different levels of accuracy, we decided to use a 10 percentile training presence as a threshold rule, which eliminates the most outlying data. We evaluated the final model using the area under the curve (AUC) values, which is a threshold-independent evaluation analysis generated by Maxent [48]. We applied the AUC classification according to Swets 1988: <0.6 invalid; 0.6–0.7 weak; 0.7–0.8 fair; 0.8–0.9 good; and 0.9–1.0 excellent. The feature selection was based on a number of occurrence data [59]. The regularization multipliers parameter was set to a default value 1.0.

To derive areas that could serve as potentially suitable for analysed representatives of the family Orthotrichaceae we used maximum training sensitivity plus a specificity-threshold value that was generated for each genus and group independently. The potential probability of occurrence was visualised on the map with a 5-degree scale with breakpoints: 0.2 (very low); 0.4 (low); 0.6 (medium); 0.8 (high); and 1.0 (very high). Probability values were reclassified by using ArcMap 10.5 software [60]. To calculate the overlap between ENMs we first transformed them to binary format using ArcMap 10.5 software [60]. Then we used SDM Toolbox v2.0 [64] to calculate the area of overlap. We depicted predicted ‘*western-montane*’ (area predicted as potentially available), ‘*eastern-lowland*’ (area predicted as potentially available), and ‘both’ (area predicted as potentially available for both studied groups) areas in the species distribution.

To compare realised ecological climatic niches of two studied groups of epiphytic mosses (‘*western-montane*’ vs. ‘*eastern-lowland*’) we conducted principal component analysis (PCA), which is a method widely used for analyzing ecological niches [65,66]. We conducted PCA on the basis of the correlation matrix using Statistica software [62]. We analysed 7 bioclimatic variables. In PCA the localities were grouped with no a priori assumptions, then marked with the symbol on the scatter plot corresponding to a particular ecoregion.

## 3. Results

### 3.1. Distribution Patterns and Ecological Preferences of Particular Genera within the Orthotrichaceae Family

MaxEnt models show excellent performance in predicting current suitable conditions for all studied genera, with an average AUC exceeding 0.9 (Table 2). In general, the bioclimatic variables connected with temperature and precipitation have the highest contributions to the models, especially bio10 and bio15, but also bio18, bio19, bio9 and bio2. However, different bioclimatic variables show the highest contributions to the models of particular genera (Table 2). Only one of the land cover variables (mixed/other trees; cons4) plays a significant role for two genera (*Leratia* and *Ulota*), whereas human footprint shows a moderate contribution to models of three genera—*Lewinskya, Orthotrichum*, and *Nyholmiella* (Table 2).

#### 3.1.1. *Leratia*

**Figure 1:**
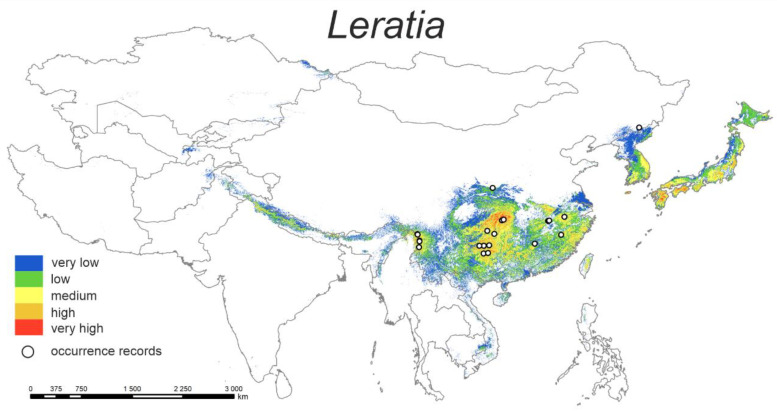
Model of the potential distribution of the genus *Leratia in China* and adjacent areas.

The predicted current distribution model (Figure 1) shows a high affinity of the species distribution with the southeastern and eastern parts of the studied area. These are primarily territories with a humid and warmer climate (e.g., SE China), or coastal regions with a mild oceanic climate (Korea, Japan).

The highest percent contributions are cons4 (48.6%) and bio19 (16.9%) (Table 2). Resulting plots of environmental conditions cons4 and bio 19 show that the probability of occurrence of genus *Leratia* increases with an increase in the percentage of land cover by mixed trees (up to 30% coverage) and when the precipitation in the coldest quarter does not exceed 500 mm.

#### 3.1.2. *Lewinskya*

**Figure 2:**
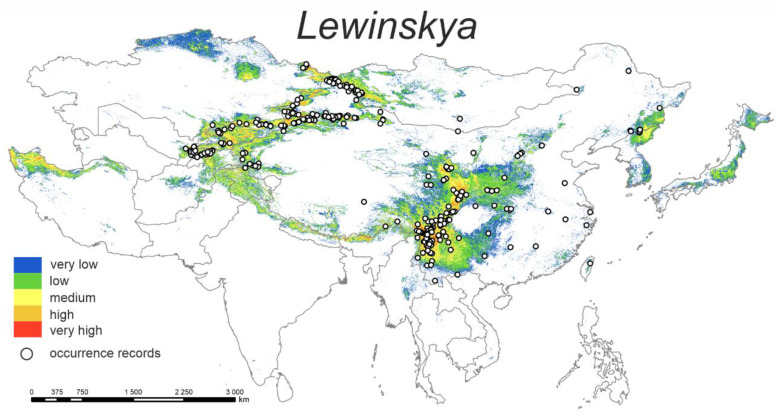
Model of the potential distribution of the genus *Lewinskya* in China and adjacent areas.

The predicted current distribution model (Figure 2) shows that the highest probability of distribution of the studied species lies in several, more or less isolated, regions of Asia. Within China, these areas are mainly found in the mountainous regions of Yunnan and Sichuan provinces. Similarly, suitable conditions are provided by the mountain massifs of Central Asia (e.g., Pamir, Tian-Shan, and Altai). Mountain valleys in the north of Iran (Elburz Mts.) or, on the contrary, in the east part of China (Changbai Mts.) also show a high affinity for species distribution.

The highest percent contributions are bio10 (40.8%) and bio15 (23.1%) (Table 2). Occurrence probability is the highest with ca. 10 up to 15 °C for mean temperature of the warmest quarter and ca. 40–80% of precipitation seasonality.

#### 3.1.3. *Macromitrium*

**Figure 3:**
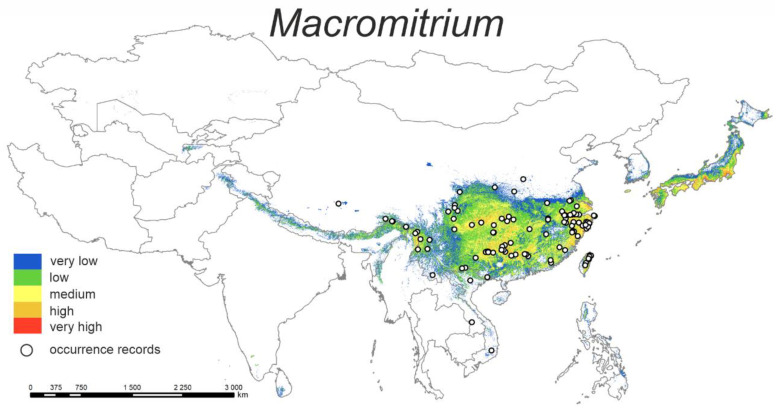
Model of the potential distribution of the genus *Macromitrium* in China and adjacent areas.

This distribution model for *Macromitrium* species (Figure 3) shows that the distribution probability of the studied species is very similar to that of the genus *Leratia* (Figure 1). The highest affinity for these species is predicted for the southeastern provinces of China, which are characterised by a warmer and more humid climate, as well as to the coastal regions in the east of China and Japan, where an oceanic climate prevails.

The highest percent contributions are bio2 (31.2%) and bio18 (27.3%) (Table 2). The probability of occurrence decreases as the mean diurnal temperature range increases (the highest with values lower than 10). In the case of bio 18, the highest probability of occurrence is when the precipitation of the warmest quarter ranges from 300 up to 1200 mm.

#### 3.1.4. *Nyholmiella*

**Figure 4:**
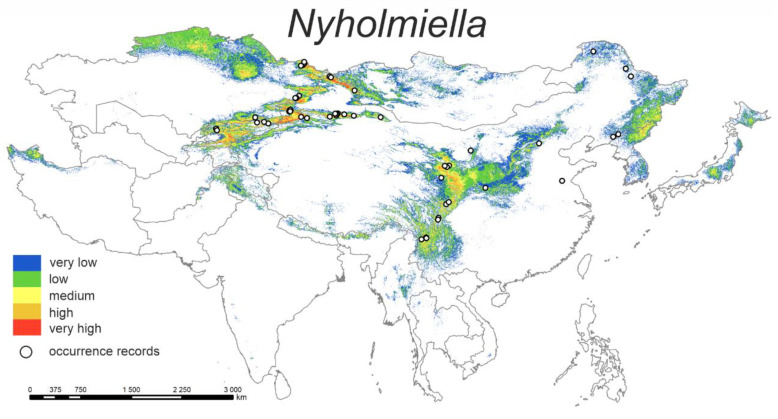
Model of the potential distribution of the genus *Nyholmiella* in China and adjacent areas.

The predicted current distribution model (Figure 4) shows that the highest probability of distribution of the studied species is mainly in the mountain ranges of northern Iran, central Asia (Tian-Shan and Altai Mts.) and especially in mountains within central Chinese provinces, e.g., Sichuan, Shaanxi, and Gansu.

The highest percent contributions are bio10 (39.7%) and bio15 (14.5%) (Table 2). Accordingly, the response curve with the highest probability of occurrence is ca. 11–16 °C of the mean temperature of the warmest quarter and ca. 40–60% of precipitation seasonality.

#### 3.1.5. *Orthotrichum*

**Figure 5:**
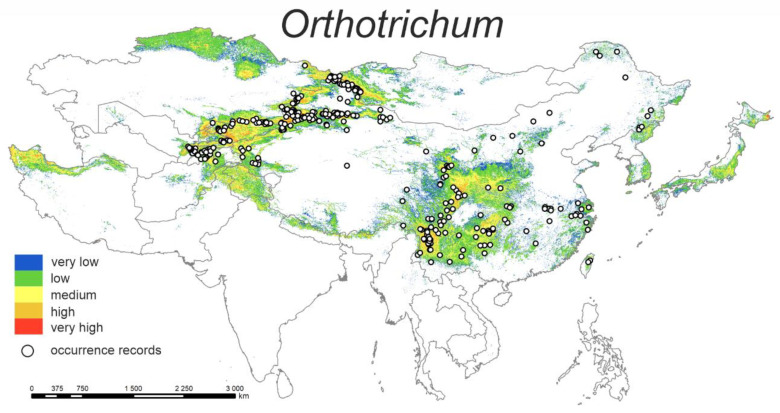
Model of the potential distribution of the genus *Orthotrichum* in China and adjacent areas.

The predicted current distribution model (Figure 5) shows that the highest probability of distribution of the studied species is in mountain ranges, e.g., Elburz Mts. (Iran), Kungey Alatau Mts. (Kyrgyzstan), Tian Shan Mts. and Altay Mts. (Xinjiang, China), Yunnan–Guizhou Plateau (China), Daba Mts. (along the Gansu-Sichuan border, China), Central Mts. and East Coast Mts. (east part of Taiwan), and the easternmost portion of the Japanese island of Hokkaidō (area of volcanoes).

The highest percent contributions are bio15 (34.6%) and bio10 (33.9%) (Table 2). Occurrence probability is the highest around 40–70% of precipitation seasonality, and ca. 10–15 °C mean temperature of the warmest quarter.

#### 3.1.6. *Ulota*

**Figure 6:**
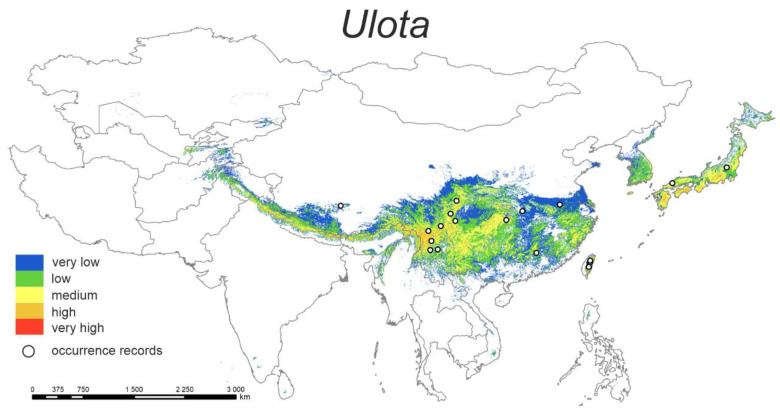
Model of the potential distribution of the genus *Ulota* in China and adjacent areas.

The predicted current distribution model (Figure 6) shows that the highest probability of distribution of the studied species is in the Himalayan range passing through India, Nepal, and Bhutan, the Yunnan–Guizhou Plateau (the north part of Yunnan province, China), south part of Sichuan province (China), south-west part of Hubei province (China), and the central mountain range (Taiwan).

The highest percent contributions are cons4 (25.5%) and bio10 (23.3%) (Table 2). Models show that the best conditions for the genus *Ulota* are when the area covered with mixed trees is higher than 30% and the mean temperature of the warmest quarter is ca. 7–16 °C.

### 3.2. Potential Distribution of Ecological Groups within Epiphytic Bryophytes

Based on climatic differences within the vast study area, we defined two ecologically distinct groups whose occurrence reflects these contrasting conditions, and prepared models of their potential distribution.

MaxEnt models produced very accurate results in predicting current suitable conditions for both studied ecological groups (*western-montane* and *eastern-lowland* epiphytes), with an average AUC exceeding 0.9 (Table 2). It was proved, that bioclimatic variables connected with temperature and precipitation have the highest contributions to the models, especially bio10, bio15, bio18, and bio9. However, the highest impact on the models of a particular group has a different set of bioclimatic variables (Table 2).

#### Potential Distribution and Niche Comparisons of ‘*western-montane*’ and ‘*eastern-lowland*’ Epiphytic Groups of Species

**Figure 7:**
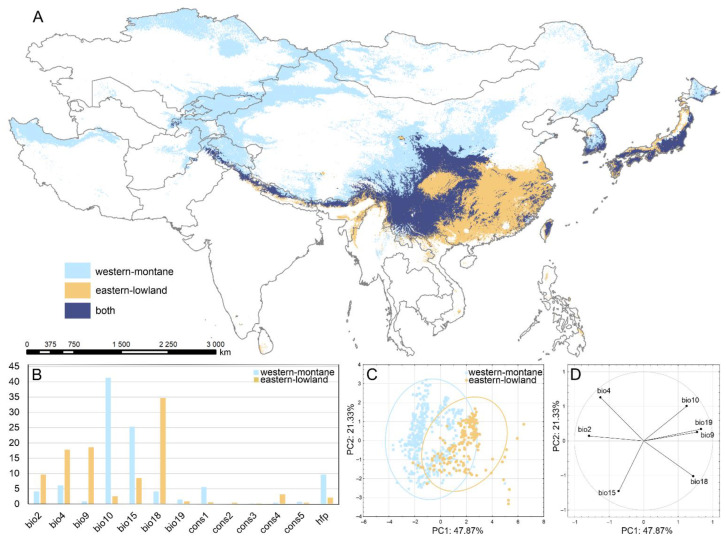
Comparison of spatial distributions and ecological niches of two studied epiphytic groups: (**A**) map of predicted potential distributions; (**B**) percent contribution of environmental variables used for ENMs; (**C**) principal component analysis (PCA) plot representing the realised niches occupied by two studied groups based on climatic variables. The ellipses represent the 95% confidence intervals; (**D**) correlations between variables and the first two principal components. For variables abbreviation see Table 1.

**Figure 8:**
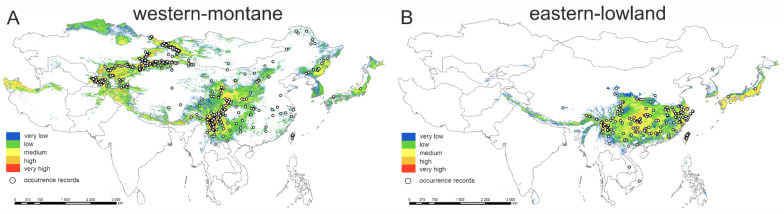
Models of potential distributions of two studied groups: (**A**) *western-montane* epiphytic group; (**B**) *eastern-lowland* epiphytic group.

The model shows the distribution patterns roughly congruent with the observed distributions for both studied groups (Figure 7A and Figure 8A,B). However, potentially suitable areas are much wider than those actually occupied, especially in the case of the *western-montane group* of epiphytic species (Figure 1, Figure 2, Figure 3, Figure 4, Figure 5, Figure 6, Figure 7A and Figure 8A,B). The area of potentially suitable habitats is almost three times wider for the *western-montane* epiphytic group than for the *eastern-lowland* one (3,805,764 km^2^ vs. 1,617,601 km^2^, respectively). In general, the eastern-lowland group occupies more south-east regions; however, both studied groups may also occupy the same geographical space (ranges overlap in an area of 1,658,565 km^2^) (Figure 7A and Figure 8A,B). For both *western-montane* and *eastern-lowland* groups of epiphytic mosses, the highest contributions to the model are bioclimatic variables, bio10 and bio15 for the *western-montane* group as well as bio9 and bio18 for the *eastern-lowland* group (Table 2, Figure 7B). Based on the current niche model occurrence probability, the *western-montane* epiphytic group has an optimum from 9 to 16 °C for mean temperature of the warmest quarter (bio10) and 40–80% of precipitation seasonality (bio15). The response curves based on the current niche model for the *eastern-lowland* epiphytic group indicate that occurrence probability is highest from about 250 mm for precipitation of the warmest quarter (bio 18) and not more than 12 °C for mean temperature of the driest quarter (bio9).

The principal component analysis (PCA), performed on seven bioclimatic variables, shows two somewhat overlapping groups of points corresponding to *western-montane* and *eastern-lowland* epiphytic groups of species (Figure 7C). The first two principal components explain over 69% of the total variance: 47.87 and 21.33% for the first and second axis, respectively. In the climate space, both studied groups differed in the first component (Figure 7C), in which the three variables (bio2, bio9, bio19), and to a lesser extent also the next two (bio10, bio18) showed high correlation. In general, species classified to the *eastern-lowland* epiphytic group occupy warmer and wettest regions than the second examined group (Figure 7C).

## 4. Discussion

The results give a useful overview of suitable habitat conditions for epiphytic bryophytes in China and adjacent areas. The potential distribution of the studied mosses largely reflects the topography as well as the distribution of climatic and vegetation zones in the studied area [31,67]. Within the Orthotrichaceae family, there can be observed two species-specific groups characterised by a difference in ecological niches linked to the climatic zones observed in eastern Asia [31,68]. The first one named here *eastern-lowland* group is related to a more humid, temperate, and subtropical climate, while the second one named *western-montane* group is related to a dryer and colder climate. Occurrence of the epiphytic mosses from the Orthotrichaceae family is predominantly influenced by climate (temperature and precipitation), which is not surprising because due to their physiology, mosses are dependent on air humidity, water availability, and drought frequency [10,69]. Similar conclusions, but on a smaller spatial scale, have been presented by Ma et al. [23] and Lou et al. [30] in modelling the habitat preferences of selected *Macromitrium* species in China. The occurrence probability of these species increased especially with increasing annual precipitation.

The occurrence of epiphytic mosses is highly related to the tree species identity as well as forest continuity [10,27,28,70]. However, in our epiphytic mosses, the tree cover (especially consensus prevalence of mixed to other trees) affects especially the distribution of *Leratia* and *Ulota*, for which occurrence probability increases along with increases in the tree cover.

Anthropopression seems to have less importance on the distribution of the studied group of mosses than we initially expected. Human footprint has a relatively small or medium impact on the occurrence of the whole family Orthotrichaceae. However, the probability of occurrence of some genera (e.g., *Lewinskya* and *Nyholmiella*) increases to a certain degree with the severity of this factor, which is associated with the planting of trees in villages and cities, as well as along roads [25,71]. Little is known about the factors that determine the distribution of epiphytic mosses in urban landscapes; however, in the last few decades, an increase in epiphytic species richness and the number of their localities in city centers were observed [72,73]. Recently a significant heat-island effect on bryophyte species growing on the ground or stones in historical Japanese moss gardens was reported by Oishi et al. [74]. Studies by Żołnierz et al. [75] also showed a significant but weak effect of this phenomenon on epiphytic mosses growing in the center of a large European city.

The results of our modelling showed a clear link between some genera and similar environmental conditions. A very similar affinity to habitat conditions was clearly demonstrated in the genera *Leratia*, *Macromitrium,* and *Ulota*. The potential distribution of the species of these genera was predicted mainly in the south and southeastern provinces of China, which are characterised by a subtropical monsoon climate [68,76]. Moreover, Yunan and Guizhou provinces are located in one of the warmest regions in China with an average daily high temperature of 29°C and high precipitation but also have a few truly tropical and sultry months [68,76]. Quite an opposite pattern in comparison to the previous three genera is clearly visible in the models of *Lewinskya*, *Nyholmiella* and *Orthotrichum*. These epiphytes prefer mainly temperate areas with continental climate often in medium-high to high mountain massifs, which are found both in west, south, and east China, as well as in other adjacent countries. These areas have a significantly cooler climate and the amount of precipitation fluctuates depending on the relief of the mountains. Slopes on the windward side collect more precipitation and, on the contrary, the leeward sides are in a rain shadow. In combination with sparser to open forest vegetation, where the spores of the epiphytic mosses can spread very effectively, these places are an ideal habitat for the occurrence of the above-mentioned genera.

One potential application of the distribution models is the forecasting of future species distribution and predicted species response to climate change [10]. The species growing in humid climates (e.g., in tropical cloud forests) seem to be more sensitive to climate change than species growing in regions characterised by recurring seasonal drought (e.g., species from seasonal lowland forests) [13]. According to Wierzcholska et al. [10], loss of mature forest ecosystems is a more important threat to woodland-specialist epiphytic bryophytes than climate change, which results from limited dispersal capabilities and specific habitat requirements. However, habitat fragmentation seems to have more disastrous effects on moisture-loving species, than on drought-resistant ones [13,77]. Thus, we can assume that representatives of the Orthotrichaceae family connected with dryer ecoregions (e.g., *Lewinskya affinis*, *L. hookeri*, *L. speciosa*, *L. vladikavkana*, *Nyholmiella obtusifolia*, *Orthotrichum alpestre*, *O. anomalum*, *O. callistomum*, *O. crenulatum*, *O. dasymitrium*, *O. pallens*, *O. pamiricum*, *O. scanicum*, *O. sordidum*, *O. urnigerum*, and *Ulota robusta*) will be more resistant to future climate warming than those from humid ones (e.g., *Leratia exigua*, *Macromitrium gymnostomum*, *M. cavaleriei*, *O. consobrinum*, *O. crispifolium*, *O. griffithii*, and *Ulota gymnostoma*). However, this phenomenon would require further research on the potential future distribution of the studied species.

Most of the Orthotrichaceae genera used in the modelling showed a strong affinity to the transition zone (ecotone), which is located vertically in the mountain areas (e.g., in the Himalayas or Hengduan Shan) and horizontally at the border of two different climate regions. This zone crosses the area of China diagonally from south to northeast, through the provinces of Yunnan, Sichuan, Shaanxi, and Heibei [31,35,36,67]. Changes that occur in population or community structures at the boundary of two or more habitats are called the ‘edge effect’ [78,79]. A consequence of the edge effect in the transition zone in China is an increase in the species diversity of epiphytic bryophytes. The conditions on the border of both climate zones favour the occurrence of both studied ecological groups (*western-montane* and *eastern-lowland* epiphytic bryophytes). Moreover, the phenomenon of a high degree of species migration within the ecotone zone has been observed [80,81], which can affect the spatial pattern of epiphytic bryophytes. The boundary of the climatic zones is therefore a clearly defined hotspot of the epiphytic species (belonging to the Orthotrichaceae family) richness in China and important areas for nature conservation purposes.

## Figures and Tables

**Table 1:** A list of environmental layers. The uncorrelated variables used for modelling are marked in bold.

Abbreviation	Name/Explanation	Unit
BIO1	annual mean temperature	degrees Celsius × 10
**BIO2**	**mean diurnal range (mean of monthly max temp—min temp)**	degrees Celsius × 10
**BIO3**	**isothermality (bio2/bio7) (×100)**	degrees Celsius × 10
BIO4	temperature seasonality (standard deviation ×100)	degrees Celsius × 10
BIO5	max temperature of warmest month	degrees Celsius × 10
**BIO6**	**min temperature of coldest month**	degrees Celsius × 10
BIO7	temperature annual range (bio5-bio6)	degrees Celsius × 10
**BIO8**	**mean temperature of wettest quarter**	degrees Celsius × 10
BIO9	mean temperature of driest quarter	degrees Celsius × 10
**BIO10**	**mean temperature of warmest quarter**	degrees Celsius × 10
**BIO11**	**mean temperature of coldest quarter**	degrees Celsius × 10
BIO12	annual precipitation	millimeters
**BIO13**	**precipitation of wettest month**	millimeters
**BIO14**	**precipitation of driest month**	millimeters
**BIO15**	**precipitation seasonality (coefficient of variation)**	percentage
BIO16	precipitation of wettest quarter	millimeters
BIO17	precipitation of driest quarter	millimeters
BIO18	precipitation of warmest quarter	millimeters
BIO19	precipitation of coldest quarter	millimeters
**hfp**	**human footprint**	percentage
**cons1**	**evergreen/deciduous needle leaf trees**	percentage
**cons2**	**evergreen broadleaf trees**	percentage
**cons3**	**deciduous broadleaf trees**	percentage
**cons4**	**mixed/other trees**	percentage
**cons5**	**shrubs**	percentage

**Table 2:** Percentage contribution of variables used for distribution modelling and performance of models measured using AUC of six genera and two studied epiphytic groups. The three highest values of the variables for each model are marked in bold.

Code	*Leratia*	*Lewinskya*	*Macromitrium*	*Nyholmiella*	*Orthotrichum*	*Ulota*	*western-montane*	*eastern-lowland*
bio10	2.60	**40.8**	1.4	**39.7**	**33.9**	9.8	**41.3**	2.6
bio15	3.30	**23.1**	3.5	**14.5**	**34.6**	1.8	**25.3**	8.5
bio18	**11.30**	2.3	**27.3**	7.9	5.1	**15.8**	4.1	**34.7**
bio19	**16.90**	1.7	1.1	4	1.5	2.8	1.5	0.9
bio2	4.40	5.6	**31.2**	4.7	3.1	5.8	4.1	9.7
bio4	0.20	6.1	9.4	3.9	**5.8**	5.4	6.1	**17.8**
bio9	5.80	1.1	**15.8**	0.3	0.7	**23.3**	0.9	**18.6**
cons1	3.40	7.4	0.7	4.5	1.2	4.5	5.6	0.6
cons2	1.40	0.3	3.2	0.2	0.2	1.6	0w.1	0.5
cons3	0.10	0.2	0.5	3.1	1	3.1	0.2	0.2
cons4	**48.60**	1	1.1	2.8	2.5	**25.5**	0.5	3.2
cons5	1.80	0.8	1.6	2.1	0.6	0.1	0.7	0.5
hfp	0.20	**9.6**	3.3	**12.5**	10	0.5	**9.7**	2.1
AUC	0.928	0.928	0.950	0.925	0.908	0.925	0.903	0.944

## Data Availability

All authors agree with MDPI Research Data Policies.
